# Genomic and physiological resilience in extreme environments are associated with a secure attachment style

**DOI:** 10.1038/s41398-020-00869-4

**Published:** 2020-06-09

**Authors:** Viviana Caputo, Maria Giuseppina Pacilli, Ivan Arisi, Tommaso Mazza, Rossella Brandi, Alice Traversa, Giampietro Casasanta, Edoardo Pisa, Michele Sonnessa, Beth Healey, Lorenzo Moggio, Mara D’Onofrio, Enrico Alleva, Simone Macrì

**Affiliations:** 1 Department of Experimental Medicine, Sapienza University of Rome, Rome, Italy; 2 Department of Political Sciences, University of Perugia, Perugia, Italy; 3 Bioinformatics, European Brain Research Institute (EBRI) Fondazione Rita Levi-Montalcini, Rome, Italy; 4 Institute of Translational Pharmacology (IFT) – CNR, Rome, Italy; 5 Bioinformatics Unit, Fondazione IRCCS Casa Sollievo della Sofferenza, San Giovanni Rotondo (FG), Italy; 6 Genomics - European Brain Research Institute (EBRI) Fondazione Rita Levi-Montalcini, Rome, Italy; 7 Laboratory of Clinical Genomics, Fondazione IRCCS Casa Sollievo della Sofferenza, San Giovanni Rotondo (FG), Italy; 8 Institute of Atmospheric Sciences and Climate, Consiglio Nazionale delle Ricerche, Rome, Italy; 9 Centre for Behavioural Sciences and Mental Health, Istituto Superiore di Sanità, Rome, Italy; 10 Biomedical Research, European Space Agency, Concordia, Antarctica; 11 Department of Physics, University of Trento, Trento, Italy

**Keywords:** Human behaviour, Physiology, Genetics

## Abstract

Understanding individual capability to adjust to protracted confinement and isolation may inform adaptive plasticity and disease vulnerability/resilience, and may have long-term implications for operations requiring prolonged presence in distant and restricted environments. Individual coping depends on many different factors encompassing psychological dispositional traits, endocrine reactivity and their underlying molecular mechanisms (e.g. gene expression). A positive view of self and others (secure attachment style) has been proposed to promote individual resilience under extreme environmental conditions. Here, we tested this hypothesis and investigated the underlying molecular mechanisms in 13 healthy volunteers confined and isolated for 12 months in a research station located 1670 km away from the south geographic pole on the Antarctic Plateau at 3233 m above sea level. Study participants, stratified for attachment style, were characterised longitudinally (before, during and after confinement) for their psychological appraisal of the stressful nature of the expedition, diurnal fluctuations in endocrine stress reactivity, and gene expression profiling (transcriptomics). Predictably, a secure attachment style was associated with reduced psychological distress and endocrine vulnerability to stress. In addition, while prolonged confinement and isolation remarkably altered overall patterns of gene expression, such alteration was largely reduced in individuals characterised by a secure attachment style. Furthermore, increased resilience was associated with a reduced expression of genes involved in energy metabolism (mitochondrial function and oxidative phosphorylation). Ultimately, our data indicate that a secure attachment style may favour individual resilience in extreme environments and that such resilience can be mapped onto identifiable molecular substrates.

## Introduction

Discovery treks, specifically those involving interplanetary spaceflights, are expected to rise steadily in the nearest future, target extremely distant locations like Mars^1^, and potentially last several months. These missions entail the participation of small groups of individuals kept in confined places, detached from their home environment, and often facing severe limitations of privacy^2^. To favour the success of the mission, it is important to minimise the potential consequences of these conditions on the well-being of crewmembers. Although few studies have been conducted in spaceflight crewmembers^3–5^, it has not yet been possible to evaluate the psychological and physiological consequences of long-term interplanetary missions in astronauts. Notwithstanding major differences in terms of gravity, the psychosocial conditions likely to occur during extended spaceflights have been mimicked on earth in prolonged isolation studies: MARS-500 and the winter-over at Concordia Station (hereafter CS). The former is a prolonged psychophysiological experiment conducted on six adult males from different nationalities. The second is an Italian-French research station, also known as white Mars (https://www.cnn.com/2015/12/09/health/white-mars-antarctica-concordia/index.html), located in Antarctica, and operating with purposes other than psychosocial experiments. Despite substantial differences, these studies indicated that prolonged confinement and isolation exert profound influences on individual physiology, psychology and behaviour^6^. Thus, the aforementioned repeated stressors may affect individual well-being, and potentially impair physiological stress reactions^7^, psychological functioning^8,9^, and neuropsychological capabilities^10^.

Individual coping with repeated stressors depends on psychological personality traits and biological predispositions. For example, the predisposition to believe that close companions will be less supportive in periods of need (high attachment anxiety) has been shown to directly relate to physiological stress (higher cortisol concentrations) and impaired immune reactivity^11^. Likewise, anxiously attached individuals are at increased risk of cardiovascular disturbances and general health problems^12–14^. Beside alterations in general physiology, these individuals show a differential pattern of brain activation when requested to predict individual behaviour with reference to concurrent mental states^15^.

Several authors demonstrated that attachment styles are moderated by genetic predisposition. For example, expression levels of genes regulating the activity of oxytocin^16^, serotonin^17^ and dopamine^18^ result in differential attachment styles. Just as genetic variants may adjust individual personality, so also social factors and environmental influences may regulate patterns of gene expression^19,20^. For example, social isolation has been associated with altered expression of genes involved in immune regulation, transcription and cell proliferation^19^.

These considerations confirm the presence of an intricate interplay between psychology, physiology and genetics in regulating how environmental adversity may impinge on individual phenotype. Understanding how these factors conspire to regulate individual resilience to extreme environments is of paramount importance to allow successful long-lasting expeditions^2,21^. To this aim, we conducted a multidisciplinary longitudinal study on 13 adult individuals that spent an average of 12 months in one of the most remote and hostile research postings in the world: Concordia Station (CS), an Italian-French outpost located at Dome C on the Antarctic Plateau at 3233 m above sea level. The extreme weather conditions experienced during winter at CS in terms of air temperature (–58 ± 9 °C^22^), altitude (barometric pressure of 478 mmHg, analogous to that experienced at 3800 above the sea level), and daylight cycle (6 months of darkness^23^) discourage outdoor excursions from the indoor research facility wherein crewmembers virtually spent all time (maximum excursion time of 2 h interspaced by recovery time into heated shelters). During the majority of the winter season, any travel to and from CS is impossible due to the extreme weather conditions. Thus, notwithstanding the presence of gravity, the other conditions (i.e. distance from home, impossibility of anticipated return, isolation, confinement, limited resources, and alterations in daylight cycle) resemble the idiosyncrasies of a prolonged manned spaceflight. A major stressor is also associated with the invariance of the crew (while during the first 3 months CS hosts up to 70 researchers/technicians, during the remaining 9 months it hosts only 13 individuals). Anecdotal reports indicate that these combined stressors may generate aberrant behaviours: let alone a homicide committed over a lost chess game in Russia’s Vostok station in 1959, a cook has been reported to voluntarily prepare “unpalatable meals” after breaking up with a co-worker (https://www.canadiangeographic.ca/article/how-antarctic-isolation-affects-mind). Within the realm of Antarctic crimes, a scientist has been accused of attempted murder for stabbing a colleague who kept spoiling the ends of books he was reading (https://www.thesun.co.uk/news/7615571/antarctic-scientist-stabs-colleague-who-kept-telling-him-endings-of-books-he-was-reading/). Beside these anecdotes, Sandal and collaborators recently reported the observation of a behavioural phenotype characterised by emotional flatness and active search of reduced stimulation: the authors referred to this behavioural constellation as “psychological hibernation”^24^.

While other studies have been conducted in Antarctica (e.g. refs. ^25–28^), this is the first one apt to investigate the psychological, physiological and molecular consequences of prolonged isolation and confinement, and potentially identify dispositional traits capable of promoting resilience towards prolonged adversity. Study participants (winter-over crewmembers, hereafter WOC) volunteered to fill questionnaires and provide biological samples (blood and saliva) before and during (30 and 150 days after arrival in Antarctica) the mission. Blood samples for gene expression analyses were also collected ~2 months after the end of the mission. Standardised questionnaires (see the “Materials and methods” section for the questionnaires utilised in this study together with corresponding references) and inventories provided information on attachment style, anxiety states and appraisal of group dynamics; salivary samples were used to determine whether the reactivity of the hypothalamic–pituitary–adrenocortical (HPA) axis (absolute values and diurnal variations in cortisol) varied as a function of attachment styles and prolonged confinement at CS; finally, whole transcriptome profiling provided relevant information regarding the biochemical and molecular pathways involved in individual adaptation to this specific stressful environment.

The multidisciplinary nature of this study, together with its unique experimental population, allowed us the rare opportunity to investigate a multifactorial response to protracted adversity (physiology and gene expression) and to identify whether a specific dispositional trait may promote resilience in extreme environments.

## Materials and methods

### Participants

Participants were 13 healthy volunteers (ten men, three women, average age 34.1 ± 3.1, range 24–56 years) from Italy, France, Switzerland and United Kingdom. Experimental procedures have been conducted in accordance with the relevant international guidelines and regulations^29,30^ and were approved by the ethical committee of the National Research Council, on 29/09/2014, decree nr. 0070015. Data collection was kept confidential by assigning each WOC a unique identification code. An informed consent was obtained from all participants prior to the beginning of the study.

### Study design

We conducted a within-participant experimental design, in which volunteers have been monitored and tested before (2 months before departure), during (30 and 150 days after arrival at Concordia), and after the winter-over (2 months after return to Europe). Baseline (before mission) and post-mission samples have been collected at the European Space Agency Centre in Cologne (Germany).

### Psychometric testing

The preliminary data collection regarding individual dispositional traits have been performed 2 months before the beginning of the winter-over and entailed the examination of participants’ attachment styles through the “Experiences in Close Relationship Scale” (ECR)^31^, a questionnaire assessing attachment anxiety and avoidance. With the exclusion of attachment styles, the data described below have also been collected 30 and 150 days after arrival at CS. Since the ECR scale provides information about a trait which, by definition and in contrast with state variables, is stable in time, we performed this evaluation only once. Conversely, state variables have been measured throughout the progress of the mission. To measure individual psychological well-being, we evaluated state anxiety^32^ and positive and negative mood through the Positive and Negative Affect Scale (PANAS)^32^. To monitor the development of group dynamics we evaluated perceived intragroup conflict (evaluating individual appraisal of task-related or interpersonal issues^33^).

### Cortisol concentrations

To study the effects of prolonged isolation and confinement on stress reactivity, we evaluated salivary cortisol concentrations 2 months before, and twice during (30 and 150 days after arrival at Concordia) the winter-over. Salivary samples were collected in cotton swabs (Salivette, Sarsted, Nümbrecht, Germany) three times a day: shortly before breakfast, at lunch and dinner. Participants were requested not to smoke, eat or drink coffee and sugary juices for 30 min before sampling. Cortisol concentrations were measured through a commercial Enzyme-Immuno-Assay kit (Salivary Cortisol ELISA (SLV-2930), DRG, Springfield, NJ, USA) according to the manufacturer’s instructions.

### Psychometric and hormonal data analysis

We preliminarily subdivided WOCs into two subpopulations characterised by secure attachment style (SAS, six WOCs) and insecure attachment style (IAS, seven WOCs) based on median values attained in the ECR scale^31^. Data obtained in these subgroups have then been analysed using a repeated measures ANOVA for split-plot designs with attachment style (SAS vs. IAS) as between participant factor and time points (pre-mission, 30 and 150 days during mission, and post-mission) as within-participant factor. With respect to salivary cortisol concentrations, we recorded few instances of missing data. While the total number of missing values was very limited (3.9%), nevertheless the dataset was partly incomplete (e.g. one participant provided all samples but the morning sample before departure and another participant provided all samples but morning and night samples 1 month after departure). Therefore, in order to provide a general evaluation of cortisol fluctuations throughout the mission and during the diurnal cycle, without interpolating missing values, we performed two separate analyses in which we compared pre-mission values once with values obtained 30 days after arrival and once with values obtained 150 days after arrival. The general statistical model for these analyses was a 2 × 2 × 3 fixed factors ANOVA: 2 (attachment style: SAS vs. IAS) × 2 (time points: pre-mission vs. 30 or vs. 150 days during mission) × 3 (diurnal variation: breakfast, lunch, and dinner). In addition, to investigate whether attachment style and cortisol reactivity were linearly related, we conducted a linear regression analysis (Spearman correlation) between absolute values of attachment style and the integral cortisol response (in the form of the area under the curve measured through the trapezoidal rule) assessed 1 and 5 months after the beginning of the mission. Due to technical reasons (samples below detection threshold), one participant had to be discarded from cortisol statistical analyses.

This experimental design allowed the analysis of the effects of the prolonged isolation and confinement on individual psychophysiology (main effect of time points), of the role of personality traits on individual psychophysiology (main effect of attachment style), and of the modulatory role of the former over the latter (interaction between attachment style and time points). ANOVA analysis was computed by the aov() function in R. Pairwise comparisons were performed either through Tukey HSD post hoc tests or in some cases, as specified in figure legends, selected pairwise contrasts tests were performed by the lsmeans() function in R-Bioconductor. Statistical significance threshold was set at *p* < 0.05.

### Sample collection and RNA extraction

Peripheral blood samples (2.5 mL) were collected in PAXgene Blood RNA Tubes (PreAnalytiX, Qiagen, Valencia, CA, USA) and stored at −80 °C before RNA extraction. Total RNA was isolated using the PAXgene Blood RNA Kit (Qiagen) according to the manufacturer’s instructions. RNA purity was assessed by using a spectrophotometer (NanoDrop ND-1000 UV-visible spectrophotometer; Labtech International, Ringmer, UK); 260/280 and 260/230 ratios were evaluated and concentrations and integrity were determined using the Agilent 2100 Bioanalyzer (Agilent Technologies, Santa Clara, CA, USA). Samples were loaded onto the Eukaryote total RNA 6000 nano Kit (Agilent Technologies, Santa Clara, CA, USA). The RNA integrity number (RIN) for all the samples was calculated and samples with a value lower than 8.0 were discarded.

### Gene expression profiling

The gene expression profiling was performed using the standard protocol for Agilent one-colour gene expression microarray. (https://www.agilent.com/cs/library/usermanuals/public/G4140-90040_GeneExpression_OneColor_6.9.pdf), using Agilent SurePrint G3 Human GE v3 8x60K chip (Design ID 072363).

### Microarray data analysis

Data extraction from the Agilent scanner images was accomplished by Feature Extraction software version 12.0. Data filtering, normalization and analysis were performed using R-Bioconductor (data are available at https://www.ncbi.nlm.nih.gov/geo/query/acc.cgi?acc=GSE149321). All the features with the flag gIsWellAboveBG=0 (too close to background) were filtered out and excluded from the following analysis steps. Filtered data were normalized by aligning samples to the 75th percentile. Differentially expressed genes were selected using R-Bioconductor by a combination of fold-change and moderated *t*-test thresholds (R-Limma test *p*-value < 0.05; |Log2 fold-change ratio|>1.0). The analysis of over- and under-represented functional gene categories and pathways was performed using the Fisher exact test, using all the array genes as “reference” genome, or through the use of Ingenuity Pathway Analysis (IPA, QIAGEN Inc.) to infer deregulated pathways and biological functions. Significantly represented pathways were presented as Forest plots and possibly accompanied by overlapping *P*-values, which measure the overlap of observed and predicted regulated gene sets, and activation Z-scores, which assess the match of observed and predicted up-/downregulation patterns and serve as both a significance measure and a predictor for the activation state of the pathways. Commonly expressed genes among time points were represented by VENN diagram. To address whether differentially expressed genes were equally distributed among experimental groups or were more represented in IAS or SAS, we performed a *χ*^2^ test. Finally, following the identification of specific altered pathways, we addressed whether these were directly associated with the attachment style. To achieve this aim, we first performed a principal component analysis of the genes expression levels contributing to the selected pathway to extract a single value (PC1, factor score) describing an individual, and then correlated this value with absolute values of attachment style (Spearman correlation) at the different time points of the mission.

## Results

### Psychometric testing

#### State anxiety

Prolonged exposure to isolation and confinement did not influence individual anxiety, whereby state anxiety remained constant throughout the mission (time points: *F*(2,18) = 0.07, *p* = 0.929). Likewise, attachment style neither influenced absolute values of anxiety (attachment style: *F*(1,9) = 1.44, *p* = 0.260; values: 28.93 ± 0.10 and 33.39 ± 2.11 for SAS and IAS, respectively) nor did it affect its progression throughout the mission (attachment style × time points: *F*(2,18) = 0.06, *p* = 0.937).

#### Positive affect

**Fig. 1:**
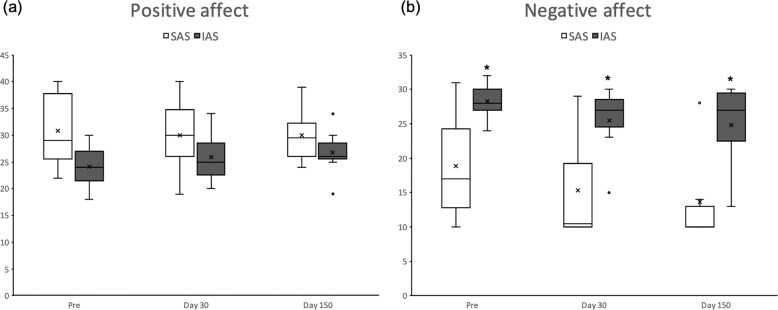
Effects of prolonged isolation and confinement on mood in SAS and IAS participants. Boxplots (median, 75th and 25th percentiles) of positive (**a**) and negative (**b**) affect as a function of mission time and attachment style. *p < 0.05 compared with SAS WOCs.

Although SAS WOCs apparently reported higher positive mood compared with IAS WOCs (32.42 ± 2.62 and 25.11 ± 2.14, respectively) such difference failed to reach statistical significance (attachment style *F*(1,8) = 4.66, *p* = 0.063). Positive mood did not vary throughout the mission (time points: *F*(2, 16) = 0.06, *p* = 0.940) and such stability was indistinguishable between SAS and IAS WOCs (attachment style × time points: *F*(2, 16) = 2.46, *p* = 0.117, see Fig. 1a).

#### Negative affect

IAS WOCs reported stronger negative mood compared with SAS individuals (attachment style: *F*(1, 8) = 9.97, *p* = 0.013; see Fig. 1b); such negative bias in IAS remained constant throughout the mission (time points: *F*(2, 16) = 0.09, *p* = 0.917).

#### Perceived group conflict scale

Compared with SAS, IAS WOCs generally reported higher scores of group conflict (attachment style: *F*(1, 9) = 3.50, *p* = 0.094; values: 1.41 ± 0.09 and 2.03 ± 0.21 for SAS and IAS, respectively) throughout the entire mission (attachment style × time points: *F*(1, 9) = 4.34, *p* = 0.067), yet these differences failed to attain statistical significance.

### Cortisol concentrations

**Fig. 2:**
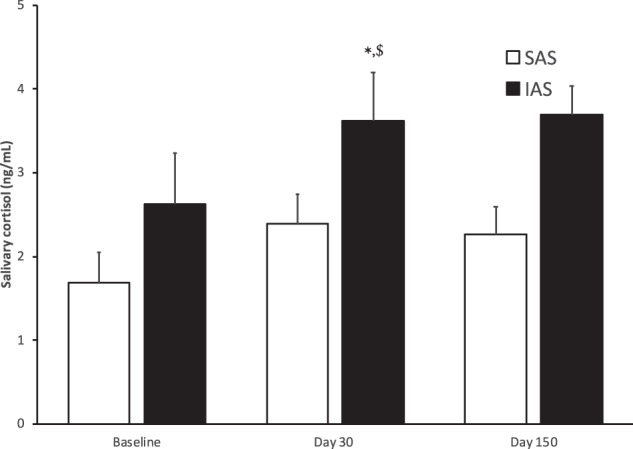
Hormonal stress reactivity to prolonged isolation and confinement in SAS and IAS participants. Average salivary cortisol concentrations (average of morning, lunch and night) at baseline, 30 and 150 days after arrival at Concordia, in secure attachment style (SAS) and insecure attachment style (IAS) WOCs. *p < 0.01 significant difference between IAS and SAS; ^$^*p* < 0.05 significantly different from baseline in the corresponding attachment style group.

In accordance with our predictions, in the absence of differences in baseline conditions, compared with IAS, SAS WOCs exhibited overall lower salivary cortisol concentrations (attachment style: *F*(1, 6) = 8.88, *p* = 0.0246, and *F*(1, 6) = 11.86, *p* = 0.0137 in the analyses comparing pre-departure vs. 30 days and pre-departure vs. 150 days after arrival, respectively, see Fig. 2). Furthermore, a month of isolation and confinement predictably increased cortisol concentrations (time points: *F*(1, 6) = 8.628, *p* = 0.026) in all WOCs, thus confirming that prolonged confinement and isolation constitute an important stressor. Post hoc analyses revealed that, while SAS and IAS volunteers did not differ in baseline conditions and 5 months after arrival at CS, SAS WOCs exhibited significantly lower salivary concentrations compared with IAS 1 month after arrival at CS. This finding strengthens the view that SAS volunteers were more resilient than IAS. Five months after the beginning of the mission, salivary cortisol concentrations were indistinguishable from those measured pre-departure (time points: *F*(1, 6) = 0.932, *p* = 0.371; see Fig. 2). Finally, the correlation analysis performed between absolute values of attachment style and cortisol area under the curve failed to indicate a linear relationship between these two factors either 1 or 5 months after the beginning of the mission (*r* = 0.075, *p* = 0.816; and *r* = 0.061, *p* = 0.859, respectively). Finally, in accordance with predictions, cortisol concentrations were higher in the morning and steadily declined to reach minimal values in the evening regardless of sampling time (diurnal variation: *F*(2, 20) = 19.771, *p* < 0.001, data not shown).

### Gene expression profiling

**Fig. 3:**
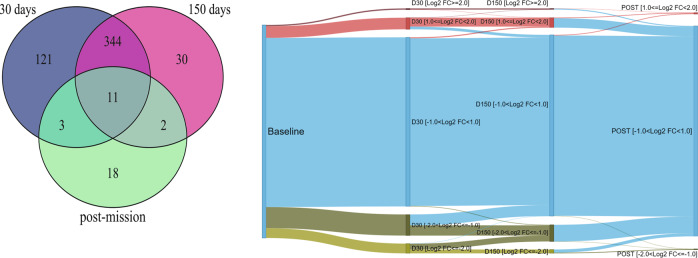
Influence of prolonged isolation and confinement on gene expression. (left panel) VENN diagram of differentially expressed genes 30 days after arrival (479 genes), after 150 days (387 genes); after 2 months from the return (34 genes); (right panel) river plot of relative expression levels. All the 15607 measured genes are shown. Log2 fold-change expression levels (Log2FC) are compared with baseline phase of the mission. Transcripts are divided into five classes, based just on Log2FC levels. Most of genes up- (red colour) or down- (green colour) regulated during the mission return to baseline level at the end of the mission. A subset of genes with Log2FC>1.0 or Log2FC<−1.0 are significantly differentially expressed. A small group of genes do not recover to the baseline level.

Prolonged exposure to confinement and isolation induced significantly different expression profiles in all volunteers. As reported in Fig. 3 (left panel), 30 and 150 days after arrival at CS, we identified 529 unique differentially expressed genes compared with the baseline conditions. The river plot (Fig. 3, right panel) shows that relative expression levels were remarkably different between baseline conditions and 30 or 150 days into mission.

While most of the genes that were up- or down-regulated during permanence at CS apparently returned to baseline levels 2 months after the end of the mission, 18 genes (see Table 1) were still deregulated. Global expression profiles (GEPs) of all WOCs measured after the trip were indistinguishable from those measured before the mission.

**Table 1:** Differentially expressed genes: post-mission vs. baseline.

Gene symbol	Description	Log2FC	*p*-value
*KLHL2*	Kelch-like family member 2	−1.41	8.15E−03
*JMJD1C*	Jumonji domain containing 1C	−1.18	1.91E−02
*SYNJ1*	Synaptojanin 1	−1.18	2.73E−02
*HIPK2*	Homeodomain interacting protein kinase 2	−1.16	4.24E−02
*USP32*	Ubiquitin specific peptidase 32	−1.11	4.75E−02
*RAB11A*	RAB11A, member RAS oncogene family	−1.11	1.14E−02
*KBTBD2*	Kelch repeat and BTB (POZ) domain containing 2	−1.03	2.55E−02
*SOAT1*	Sterol O-acyltransferase 1	−1.02	4.07E−02
*PTPN12*	Protein tyrosine phosphatase, non-receptor type 12	−1.02	1.08E−02
*RNU12*	RNA, U12 small nuclear	1.06	4.96E−02
*SNORD22*	Small nucleolar RNA, C/D box 22	1.09	3.71E−02
*SNORA46*	Small nucleolar RNA, H/ACA box 46	1.14	3.60E−02
*SNORD83B*	Small nucleolar RNA, C/D box 83B	1.21	4.97E−02
*SCARNA16*	Small Cajal body-specific RNA 16	1.27	4.00E−02
*SNORD74*	Small nucleolar RNA, C/D box 74	1.30	3.60E−02
*SNORA2B*	Small nucleolar RNA, H/ACA box 2B	1.33	1.66E−02
*SNORA21*	Small nucleolar RNA, H/ACA box 21	1.34	1.86E−02
*SNORA28*	Small nucleolar RNA, H/ACA box 28	1.95	3.44E−02

List of differentially expressed genes in the comparison between gene expression profiles (GEP) conducted after the mission and at baseline in insecurely attached participants.

**Fig. 4:**
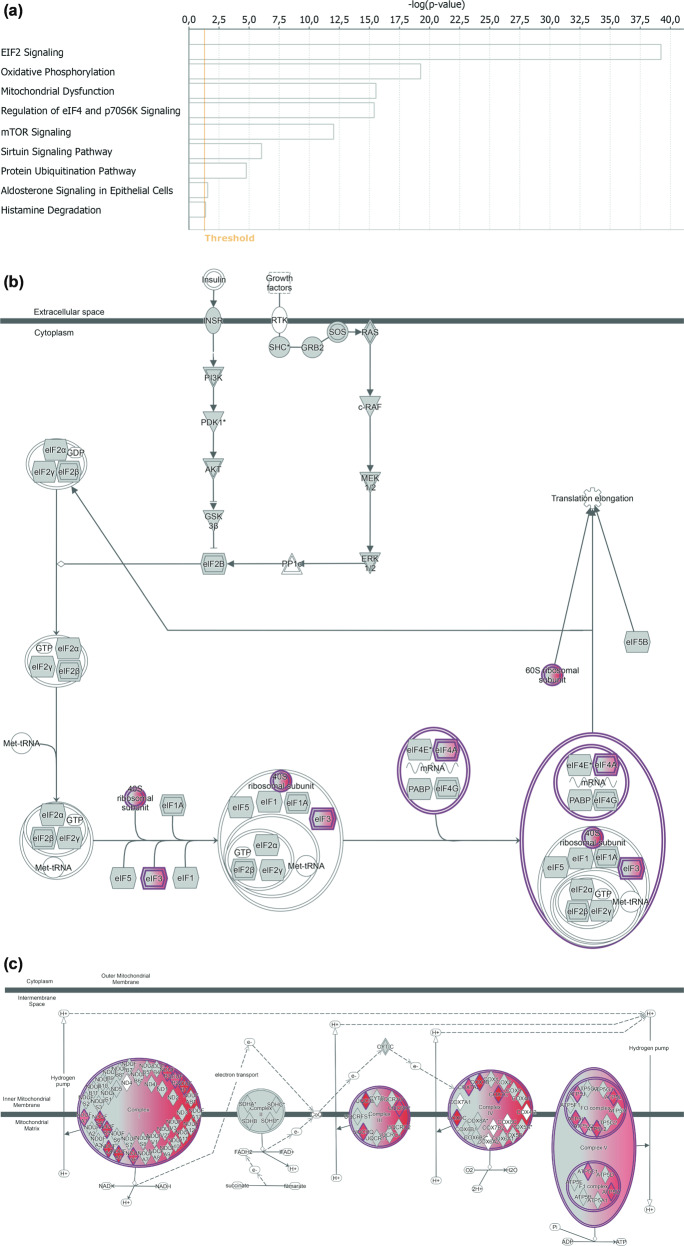
Gene enrichment analysis and desription of relevant deregulated pathways. **a** Significantly deregulated pathways 30 days after arrival. The significance threshold is set to −log10(*p*-value)=1.3; **b** “EIF2”; and **c** “Oxidative phosphorylation” signalling pathways deregulated during the whole mission. Marked in red all genes differentially expressed already after 30 days.

Enrichment analyses of genes differentially expressed 30 days after arrival (Fig. 4a) showed that the deregulated pathways were related to protein synthesis (“EIF2 signalling pathway” and “Regulation of eIF4 and of p70S6K signalling”, Fig. 4b), mitochondrial function (“Oxidative Phosphorylation” and “Mitochondrial dysfunction”, Fig. 4c), and further pathways, i.e. “mTOR”, “Sirtuin” and “Protein ubiquitination” signalling pathways.

The dysregulation observed in these pathways was consistent throughout the mission and apparently returned to baseline conditions 2 months after the end of the mission. Predictably, one of the earliest biological functions to be significantly deregulated (30 days after arrival, and not 150 days after arrival) was “Circadian rhythm” (*p* = 0.0292), for which we observed 10 deregulated genes: *DYRK1A*, *FAS*, *FBXL3*, *NR1D2*, *NRIP1*, *PPP1CB*, *PRKAA1*, *PRNP*, *SIRT1*, *UBE3A*. In addition, in accordance with our predictions, we observed that the winter-over at CS also altered immune system functions, in terms of hyperactivation of the immune response. Specifically, we observed that the following representative processes were significantly enriched throughout the entire period: cell-to-cell signalling and interaction, humoral immune response, haematological system development and function, immune cell trafficking, and inflammatory response (*p* < 3.09E−02).

**Fig. 5:**
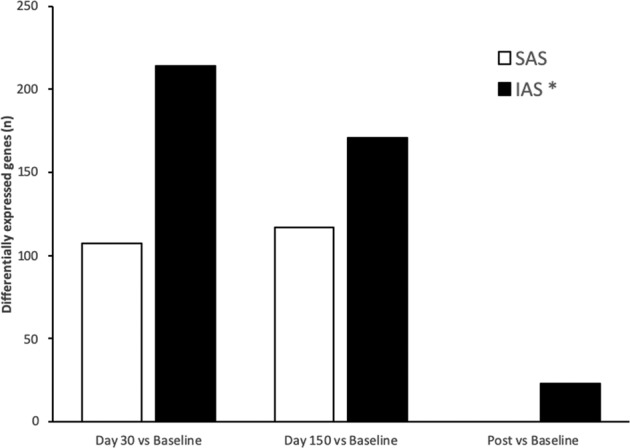
Differential patterns of gene expression in response to prolonged isolation and confinement in SAS and IAS participants. Proportion of differentially expressed genes between baseline conditions and day 30, day 150 and post-mission in securely attached participants (SAS) and insecurely attached participants (IAS). *p < 0.01 in the comparison between SAS and IAS subjects. Histograms have been obtained combining genes from “Oxidative phosphorylation”, “Mitochondrial” and “Sirtuin” pathways.

Factoring attachment style into the analysis (R-Limma linear model, see Materials and methods) further clarified the overall GEP patterns. First, we observed that the overall number of differentially expressed genes (DEGs) increased; second, the proportion of DEGs was much higher in IAS than in SAS WOCs, thus suggesting that the latter are more resilient than the former. Specifically, the proportion of DEGs between the members of the two groups were significantly different for the following comparisons: D30 vs. baseline (*p* < 0.001), D150 vs. baseline (*p* < 0.001), and post-mission vs. baseline (*p* < 0.001). Just as the proportion of DEGs resulted increased, so also the corresponding pathways resulted more enriched of DEGs when dispositional traits were included in the analysis. Thus, while the affected pathways remained constant regardless of the inclusion of attachment style, the number of DEGs varied in the two groups. For example, the “oxidative phosphorylation” pathway resulted active in both groups, albeit at different rates: IAS WOCs expressed more genes belonging to this pathway (37 (D30) and 22 (D150)) than SAS (18 (D30) and 20 (D150)). Likewise, concerning the “mitochondrial dysfunction” pathway, IAS expressed 42 (D30) and 32 (D150) genes, while SAS expressed 19 (D30) and 23 (D150) genes. Finally, the “Sirtuin signalling” pathway was represented by 55 (D30) and 45 (D150) genes in IAS and by 25 (D30) and 28 (D150) genes in SAS. Furthermore, as shown in Fig. 5, the GEPs of the aforementioned pathways had a differential pattern in the two subgroups, whereby the number of GEPs altered at any given time point was higher in IAS than in SAS volunteers (*χ*^2^(2) = 14.24, *p* < 0.001). The specific genes pertaining to the aforementioned pathways have been reported in the supplementary information (Table 1 SI). Finally, we did not observe a linear relationship between these alterations and dispositional traits, as reported in the supplementary information (Table 2 SI).

In addition, while the dysregulation profile was constant at D30 and at D150, and then normalized after the mission in SAS individuals, it followed a differential course in IAS WOCs. This group attained peak values at D30, and then exhibited a steady decline until the end of the mission. Importantly, compared with baseline conditions, IAS individuals showed an altered GEP of the relevant pathways at day 30 (*p* < 0.001; 95% CI: 23.2–37.14%), day 150 (*p* < 0.001; 95% CI: 8.15–22.43%) and after the end of the mission (*p* < 0.001; 95% CI: 4.11–9.57%), thus indicating that, in contrast with SAS WOCs, they failed to fully recover from the extreme circumstances experienced at CS.

## Discussion

The present study demonstrates that securely attached individuals have increased resilience under extreme circumstances, and that such resilience can be anchored onto identifiable molecular substrates. Unsurprisingly, the mission at CS represented a major stressor whereby it affected HPA reactivity and significantly altered gene expression. Yet, while we expected dispositional traits to mediate individual vulnerability/resilience, we did not anticipate the relationship between an easily identifiable attachment style and resilience to be so pervasive: secure attachment resulted in a more confident appraisal of the mission (reduced negative mood and perceived group conflict), reduced HPA axis susceptibility, and even less pronounced alteration of gene expression profiles.

In accordance with our predictions^6^, prolonged isolation and confinement resulted in a transient increase in salivary cortisol concentrations. Jacubowsky and collaborators reported that morning salivary cortisol concentrations were significantly increased by confinement and isolation in the MARS-500 experiment^34^. In contrast with this finding, Feuerecker and collaborators reported that 1 month of permanence at CS did not alter absolute concentrations and diurnal patterns of salivary cortisol secretion^28^. We believe that this apparent conflict may be related to the fact that in the study of Feuerecker and colleagues^28^ participants were not stratified for dispositional traits. Here, the increase in salivary cortisol concentrations, apparently pertaining to the overall study population, was significant only in IAS and not in SAS volunteers. Accordingly, Pietromonaco and colleagues observed that insecure attachment style related to increased cortisol reactivity to a psychological stressor^35^. Importantly, 5 months after arrival at Concordia, absolute cortisol concentrations were indistinguishable from baseline conditions, thus suggesting that the effects of the mission on stress-related hormones were transient.

Psychological reactions to the mission were also modulated by dispositional traits whereby IAS participants exhibited increased negative mood than SAS counterparts. The interdependency between attachment style and negative mood has already been observed both in depressed patients and healthy controls^36,37^. Predictably^38^, the increase in negative mood also reverberated in the fact that, compared with SAS, IAS WOCs seemed more concerned by potential conflicts within the group. Ultimately, endocrine and psychometric variables indicate that prolonged confinement and isolation represented a remarkable, yet transient, stressor to the study population and that a secure attachment style considerably mitigated its consequences. It is important to notice that the transient nature of the protracted isolation may be partly confounded by the small sample size adopted in this study. While the living conditions of the WOCs allowed the possibility to conduct an experiment with a remarkable degree of standardization, the limited number of participants may have marginally reduced statistical power thereby potentially masking biological effects characterised by small effect sizes. Future studies are needed to increase the sample size.

These results were paralleled by underlying molecular mechanisms. Thus, while confinement and isolation remarkably, yet transiently, affected gene expression, a secure attachment style related to reduced perturbation. The association between psychological factors and alterations in gene expression have already been reported in several studies^19,39–41^ conducted both in humans and laboratory animal species^42,43^. For example, Cole and collaborators^19^ demonstrated that social isolation altered the expression of genes involved in immune regulation. Beside psychological factors, also physical environmental influences like radiations^44^, altitude^45,46^, and hypoxia^20^ have been repeatedly reported to alter gene expression.

While alterations in gene expression were expected, their specificity and selectivity are particularly enticing. The mechanisms and biological processes primarily affected revolved around mitochondrial function, protein synthesis, immune response and circadian rhythms. The alteration in mitochondrial function may reflect individual adaptation to the protracted hypoxia experienced at Concordia^26^. Porcelli and collaborators^26^ demonstrated that the prolonged hypoxic stress experienced at CS persistently elevates blood haemoglobin, carbon dioxide partial pressure and arterial oxygen saturation. In accordance with its modulatory role in the respiratory chain (oxygen consumption and production of reactive oxygen species)^47^ mitochondrial function has been shown to vary in response to protracted hypoxia^48^. Thus, the alteration in mitochondria-related gene expression profiles may represent one of the molecular substrates of the respiratory adaptations occurring at Concordia. A complementary aspect, potentially contributing to alterations in mitochondria, relates to dietary patterns. Thus, several authors demonstrated that variations in diet may translate into alterations in mitochondrial function^49,50^. While we cannot exclude that the diet played a role in the observed alterations, we suggest that this factor may not represent the most likely candidate. All WOCs had access to the same diet throughout the entire mission, which was daily prepared by the chef of the station (an Italian cook with training in French and Italian cuisine). The diet was constituted by a huge diversity of food and was very similar to that consumed while in Europe. Thus, although it is not possible to exclude that this factor played a role, we would consider its influence potentially marginal.

Beside physiology, mitochondria may also mediate the psychological appraisal of the mission. Specifically, mitochondria have been proposed as the molecular fingerprint of individual stress reactivity^51^. Such reactivity encompasses short- and long-term adaptations (e.g. increase in heart rate^52^) to both acute and chronic challenges^53^. Importantly, beside mediating catecholaminergic responses, mitochondria are also responsible for the synthesis and metabolism of glucocorticoids^54^, thereby exerting profound effects on the overall machinery underlying adaptive responses to physical and psychological challenges^55–58^. It is thus tenable that the alterations in mitochondrial gene expression are partly responsible for the individual reaction to the protracted stressors of different nature encountered at Concordia.

The alterations in the protein synthesis pathways likely reflect the general adaptation of the organism to the altered psychosocial and environmental conditions. Specifically, both psychological and environmental adversities may have altered the function of ribosomes, the primary site of the molecular machinery of protein synthesis^59,60^. Our results are in accordance with available literature indicating that several environmental variables like diet, hypoxia and radiations are capable of modulating ribosome expression and function^20,61^. Just as physical challenges may influence the function of mitochondria and ribosomes, so also psychological stressors have been shown to interfere with energy metabolism^51^ and protein synthesis^62^. Social hierarchies, in the form of protracted social defeat have been shown to modulate ribosomal activity also in laboratory animals^63^. The combination of the physiological and psychological challenges experienced at CS may also explain the observed alterations at the level of the immune system^11,28,64,65^. Thus, factors like altitude, hypoxia, social isolation and fear of the unknown in Antarctica have been suggested to influence the activity of the immune system^65^. Finally, the finding that, regardless of dispositional traits, study participants exhibited an overall variation in the expression of genes associated with circadian rhythms is likely related to the fact that the familiar light-dark cycle at CS was displaced by 6 months of constant darkness.

Just as dispositional traits mitigated the effects of the mission on psychoneuroendocrine measurements, so also they were associated with a reduced susceptibility at the level of gene expression, a candidate biological mediator of resilience in extreme environments. In particular, we suggest that the increased resilience observed in SAS individuals is explained by their reduced susceptibility in gene expression at the level of mitochondria^51^. As mentioned above, beside orchestrating the synthesis and metabolism of the hormones involved in stress reactivity, the functionality of these organelles is strongly affected by external challenges. Furthermore, mitochondria mediate individual bodily functions like sleep-wake cycles and appetite, cognitive processes like memory and attention, and social behaviour (see ref. ^51^ for a review). Thus, while this hypothesis warrants further experimental support, the differential sensitivity in mitochondrial gene expression observed in SAS individuals may represent the molecular fingerprint of their psychoneuroendocrine resilience.

Ultimately, the present study indicates that a dispositional trait rapidly identified through a standardised test battery may represent a fundamental aid in the selection of the personnel involved in missions entailing protracted isolation and confinement of a restricted group of individuals.

## Supplementary information

Table 1 SI

Table 2 SI
