# Supplementary material for: Genomic and physiological resilience in extreme environments are associated with a secure attachment style
Source: Transl Psychiatry. 2020 Jun 9;10:185. doi: 10.1038/s41398-020-00869-4 (PMC7283351; doi:10.1038/s41398-020-00869-4)
Supplement: Supplementary file 2 — Table 2 SI [file 41398_2020_869_MOESM2_ESM.docx]

**Table 2 SI. Correlational analyses between attachment style and selected gene pathways**

|  | Baseline | | One month | | Five months | | Post-mission | |
| --- | --- | --- | --- | --- | --- | --- | --- | --- |
| Gene pathway | r value | p value | r value | p value | r value | p value | r value | p value |
| Oxidative phosphorilation | 0.005 | 0.99 | -0.052 | 0,86 | 0.260 | 0.41 | -0.080 | 0.79 |
| Mitochondrial Dysfunction | -0.032 | 0.93 | -0.019 | 0,95 | 0.214 | 0.50 | -0.080 | 0.79 |
| Sirtuin signalling | -0.041 | 0.90 | -0.033 | 0,91 | -0.035 | 0.91 | -0.102 | 0.74 |
| EIF2 signalling | 0.214 | 0.53 | 0.005 | 0,99 | -0.109 | 0.74 | -0.254 | 0.40 |

Correlational analysis (Spearman) between attachment style (absolute values attained in the ECR scale) and alterations in the expression of the genes contributing to the corresponding pathways (factor score following a principal component analysis: the PC1 was selected) observed in the different time points investigated in the present study (baseline, one month, five months, post-mission). In the table we present the gene pathway (left column), the Spearman correlation value (centre column), and the corresponding p value (right column).
